# Clinical Manifestations and Molecular Diagnosis of Scrub Typhus and Murine Typhus, Vietnam, 2015–2017

**DOI:** 10.3201/eid2504.180691

**Published:** 2019-04

**Authors:** Nguyen Vu Trung, Le Thi Hoi, Vu Minh Dien, Dang Thi Huong, Tran Mai Hoa, Vu Ngoc Lien, Phan Van Luan, Sonia Odette Lewycka, Marc Choisy, Juliet E. Bryant, Behzad Nadjm, H. Rogier van Doorn, Allen L. Richards, Nguyen Van Kinh

**Affiliations:** National Hospital for Tropical Diseases, Hanoi, Vietnam (N.V. Trung, L.T. Hoi, V.M. Dien, D.T. Huong, T.M. Hoa, V.N. Lien, N.V. Kinh);; Hanoi Medical University, Hanoi (N.V. Trung, L.T. Hoi, P.V. Luan, N.V. Kinh);; Oxford University Clinical Research Unit and Wellcome Trust Major Overseas Programme, Hanoi (S.O. Lewycka, M. Choisy, J.E. Bryant, B. Nadjm, H.R. van Doorn);; University of Oxford, UK (S.O. Lewycka, B. Nadjm, H.R. van Doorn);; Institute of Research for Development, Marseille, France (M. Choisy); Merieux Foundation, Lyon, France (J.E. Bryant);; Naval Medical Research Center, Silver Spring, Maryland, USA (A.L. Richards);; Uniformed Services University of the Health Sciences, Bethesda, Maryland, USA (A.L. Richards)

**Keywords:** Scrub typhus, murine typhus, rickettsial diseases, bacteria, clinical manifestations, molecular diagnosis, Vietnam

## Abstract

Rickettsioses are endemic to Vietnam; however, only a limited number of clinical studies have been performed on these vectorborne bacteria. We conducted a prospective hospital-based study at 2 national referral hospitals in Hanoi to describe the clinical characteristics of scrub typhus and murine typhus in northern Vietnam and to assess the diagnostic applicability of quantitative real-time PCR assays to diagnose rickettsial diseases. We enrolled 302 patients with acute undifferentiated fever and clinically suspected rickettsiosis during March 2015–March 2017. We used a standardized case report form to collect clinical information and laboratory results at the time of admission and during treatment. We confirmed scrub typhus in 103 (34.1%) patients and murine typhus in 12 (3.3%) patients. These results highlight the need for increased emphasis on training for healthcare providers for earlier recognition, prevention, and treatment of rickettsial diseases in Vietnam.

## Abstract 2

Scrub typhus was the predominant rickettsial disease diagnosed among hospitalized patients with acute undifferentiated fever in northern Vietnam.

Rickettsioses are vectorborne infections caused by small, gram-negative, obligatory intracellular bacteria of the genera *Rickettsia* and *Orientia*, belonging to the family *Rickettsiaceae*. The most common vectors for *Rickettsiaceae* are fleas, mites, ticks, and lice. The pathogens are classified into 3 groups on the basis of clinical characteristics of disease and antigenicity: scrub typhus group orientiae (STGO), spotted fever group rickettsiae (SFGR), and typhus group rickettsiae (TGR) (*1*–*3*). The presence and distribution of *Rickettsia* and *Orientia* spp. agents in Southeast Asia are poorly defined (*4*–*6*). Additional surveillance studies are needed to improve diagnosis and patient treatment.

The extent and severity of disease associated with scrub and murine typhus in Vietnam is unknown because both clinical- and laboratory-based diagnostics are limited and no systematic surveillance programs or reporting systems are in place (*7*). Frequent cases of scrub typhus and murine typhus were reported among US military personnel stationed in Vietnam during the 1970s (*6*–*8*). Cases also have been identified among returning travelers from the region (*9*,*10*). One recent study demonstrated that patients with acute undifferentiated fever admitted to a large referral hospital in Hanoi were often infected with scrub typhus (40.9%) and murine typhus (33.3%) (*11*). A previous study in the same hospital showed that scrub typhus accounted for 3.5% (251/7,226) of all admissions and 33.5% (251/749) of clinical admissions that met the clinical criteria for testing to the infectious diseases department (*12*). Another clinical study conducted in central Vietnam reported the use of eschar swabbing for molecular diagnosis and genotyping of *O. tsutsugamushi*, which appeared helpful for rapid case detection in the early stage of infection (*13*). Together, these findings strongly suggest that rickettsioses are common causes of acute fever in Vietnam that are often underdiagnosed and undertreated.

We conducted a prospective hospital-based study of clinically suspected rickettsiosis in the National Hospital for Tropical Diseases (NHTD) and Bach Mai Hospital, 2 large tertiary-care referral hospitals in Hanoi, during 2015–2017. We describe the clinical characteristics of the cases and the assessment of molecular testing results for diagnosing scrub typhus and murine typhus.

## Methods

### Study Design

We conducted a prospective observational study of clinically suspected rickettsioses at NHTD and Bach Mai Hospital. We included patients ≥15 years of age with fever (temperature >37.5°C) of unknown cause or presence of ≥1 eschar and ≥1 of the following clinical symptoms: rash, headache, myalgia, lymphadenopathy, hepatomegaly, or splenomegaly. We excluded patients with laboratory confirmed malaria, dengue, measles, or influenza (via blood smear or rapid tests); patients with clinically and radiologically confirmed pneumonia; and patients with microbiologically confirmed sepsis or urinary tract infection.

We recorded information regarding patient demographics, medical history, clinical and laboratory findings, and treatment in a case record form. We classified outcome at discharge as full recovery, death, or palliative discharge. Palliative discharge is a commonly preferred alternative to dying in the hospital in Vietnam, where a patient for whom ongoing care is considered futile is discharged to permit them to die in their home with their family.

### Ethics Statement

We obtained written informed consent from patients ≥18 years of age and from patients and their parents or guardians for those 15–17 years of age enrolled at admission. The study protocol was approved by the institutional review boards of NHTD (document no. 01A/HDDD-NDTU) and Bach Mai Hospital (document no. 6/BM-HDDD).

### Specimen Collection and Laboratory Testing

Blood was drawn from all patients at admission for blood culture and basic laboratory tests. These tests included complete blood counts, biochemistry, procalcitonin, and serologic and molecular testing for rickettsioses.

### Serologic Analysis 

We screened serum samples at 1:100 dilution and considered net optical density of >0.5 positive, as described (*14*,*15*). ELISA antigen preparations were derived from *R. typhi* Wilmington for TGR-specific IgG, *R. conorii* Malish 7 for SFGR-specific IgG, and *O*. *tsutsugamushi* Karp, Kato, and Gilliam for the detection of STGO-specific IgG.

### Quantitative Real-Time PCR 

We collected whole blood in EDTA tubes, then processed it with Ficoll-Paque to obtain leukocyte buffy coats. We extracted DNA from the buffy coats using QIAamp DNA Mini Kits (QIAGEN, https://www.qiagen.com) according to the manufacturer’s instructions and previously described assays (*16*–*19*). Diagnosis of scrub typhus was based on species-specific quantitative real-time PCRs (qPCRs) targeting the corresponding 47-kDa outer membrane protein gene of *O. tsutsugamushi* (Otsu47) and murine typhus was diagnosed based on the outer membrane protein B gene of *Rickettsia typhi* (Rtyph). When both species-specific assays were negative but the genus-specific qPCR assay targeting the17-kDa antigen gene (Rick17b) was positive, we classified the diagnoses as nonscrub typhus-nonmurine typhus rickettsiosis. We initially tested all buffy coat samples in this study with the Otsu47 qPCR assay to detect *O. tsutsugamushi*. We subsequently assessed Otsu47-negative samples with the genus-specific qPCR assay Rick17b to detect *Rickettsia* spp. We subsequently tested Rick17b-positive samples with the Rtyph qPCR. For each qPCR, we extracted 1 μL of nucleic acid from the buffy coats and added it to a final reaction volume of 25-μL, using Platinum Quantitative PCR SuperMix-UDG (Invitrogen, https://www.thermofisher.com), and performed real-time PCR on an Applied Biosystems 7500 Fast Real-Time PCR System (Applied Biosystems, https://www.appliedbiosystems.com).

### Statistical Analysis

We summarized categorical variables as frequencies and percentages and used the Fisher exact test to compare clinical characteristics among different groups. We summarized non–normally distributed continuous variables as medians with interquartile ranges (IQRs) and compared them using the Mann-Whitney test. We summarized data following normal distribution as means with SDs and compared them using the analysis of variance test for 2-group comparisons. To investigate whether cases from outside the province of Hanoi were biased toward higher severity, we tested the differences in symptoms between Hanoi and the other provinces using the Fisher exact test, with Benjamini and Hochberg correction for multiple comparisons (*20*). We performed analyses using Stata 12.0 (StataCorp LLC, https://www.stata.com).

## Results

### Patient Population and Enrollment

**Figure 1:**
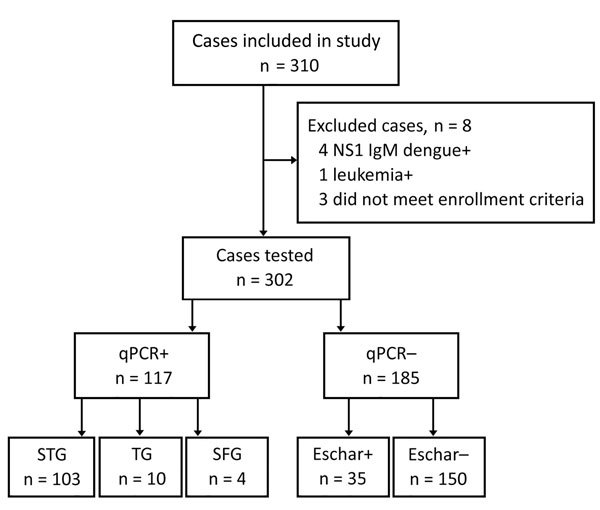
Enrollment flow, qPCR results, and presence of eschar among enrollees in study of rickettsial patients, Vietnam, March 2015–March 2017. NS1, nonstructural protein 1; qPCR, quantitative PCR; SFG, scrub typhus group; SFG, spotted fever group; STG, scrub typhus group; +, positive; –, negative.

**Figure 2:**
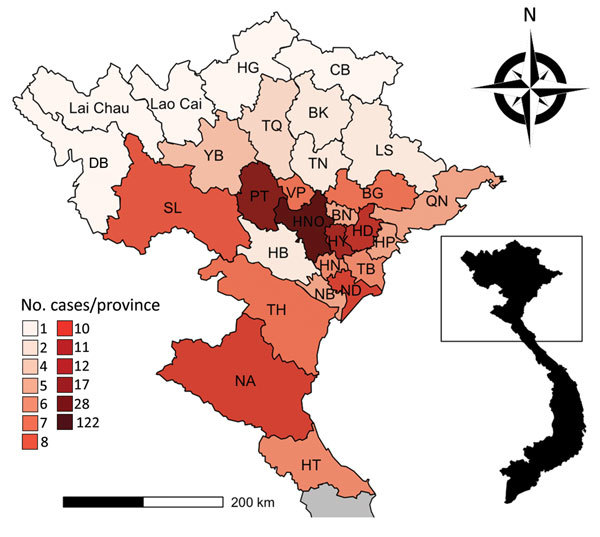
Distribution of 302 rickettsial patients, by province, in northern Vietnam, March 2015–March 2017. A) Provinces of northern Vietnam; inset shows location within Vietnam. B) Number of cases by province. BG, Bac Giang; BK, Bac Kan; BN, Bac Ninh; CB, Cao Bang; DB, Dien Bien; HG, Ha Giang; HB, Hoa Binh; HD, Hai Duong; HN, Ha Nam; HP, Hai Phong; HT, Ha Tinh; HY, Hung Yen; LS, Lang Son; NA, Nghe An; NB, Ninh Binh; ND, Nam Dinh; NHO, Hanoi; PT, Phu Tho; QN, Quang Ninh; SL, Son La; TB, Thai Binh; TH, Thanh Hoa; TN, Thai Nguyen; TQ, Tuyen Quang; VP, Vinh Phuc; YB, Yen Bai.

We enrolled 310 persons with suspected rickettsiosis cases during March 2015–March 2017. Eight cases were excluded because diagnostic assays showed positive results for other infections (Figure 1). The median age of the final study population (n = 302) was 50 years (IQR 36–60); 158 (52.3%) were men and 144 (47.7%) women. The 302 patients originated from 28 northern provinces. The largest proportion came from the provinces of Hanoi (122, 40.4%), PhuTho (28, 9.3%), and HungYen (17, 5.6%) (Figure 2). Ninety-seven (32.1%) patients were admitted directly from the community to the study hospitals and 205 (67.9%) were referred from other hospitals. Of the 205 transferred patients, 134 (65.4%) were from hospitals outside Hanoi (Table 1). Symptoms did not differ between those admitted in Hanoi and those transferred from other provinces (Table 2).

**Table 1:** Demographic characteristics for 302 patients enrolled in a study of rickettsial diseases, northern Vietnam, March 2015–March 2017*

Characteristic	Value
Age, y, median (IQR), range	50 (36–60), 16–89
Sex	
M	158 (52.3)
F	144 (47.7)
Admitted from community	97 (32.1)
Transferred from other hospital or clinic	205 (67.9)
Received antimicrobial drugs before admission	83 (27.5)
Residence in rural area	171 (56.6)
Farmer	127 (42.1)

*Values are no. (%) patients except as indicated. IQR, interquartile range.

**Table 2:** Comparison of signs and symptoms for patients with scrub typhus from Hanoi Province with patients from other provinces, northern Vietnam, March 2015–March 2017*

Sign or symptom	p value	
	Noncorrected	Corrected
Headache	0.7041	0.9071
Myalgia	0.8773	0.9071
Eye pain	0.7745	0.9071
Nausea	0.7295	0.9071
Vomiting	0.5109	0.9071
Diarrhea	0.8530	0.9071
Abdominal pain	0.2053	0.6676
Throat pain	0.8128	0.9071
Cough	0.0158	0.2194
Rash	0.8683	0.9071
Eschar	0.0219	0.2194
Skin hyperemia	0.3239	0.8098
Congestive conjuctiva	0.9071	0.9071
Lymphadenopathy	0.0590	0.3479
Edema	0.0696	0.3479
Rales	0.6423	0.9071
Resonance from lungs	0.5542	0.9071
Liver enlargement	0.2089	0.6676
Spleen enlargement	0.5725	0.9071
Convulsions	0.2336	0.6676

*n = 115; results use Fisher exact test, expressed in p values and corrected using multiple comparisons and the Benjamini and Hochberg method (*20*).

**Figure 3:**
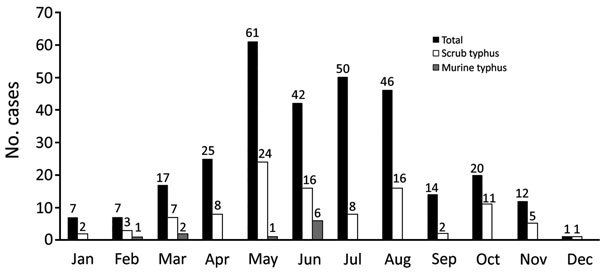
Frequency of all enrolled patients and those with confirmed scrub typhus or murine typhus in National Hospital for Tropical Diseases and Bach Mai Hospital, by month, Vietnam, March 2015–March 2017.

Of the 302 patients, 171 (56.6%) reported living in rural areas; 127 (42.1%) also reported that they were farmers. During the study, 224 (74.2%) patients were admitted to the hospital in the summer (April–August), with the highest number (61, 20.2%) in the month of May (Figure 3). The median delay in the time from onset of symptoms to treatment was 9 days (range 6–11 days). Eight patients were admitted to the study ≥30 days after onset of symptoms, 1 of whom was admitted 41 days after symptoms began; all 8 were successfully treated. Furthermore, 83 (27.5%) patients reported use of antimicrobial drugs before initial admission to the hospital, although the identity of these drugs was not given.

We assessed admission serum samples for IgG against STGO, TGR, and SFGR using ELISAs in a subset of 121 of the 302 enrolled patients. Of these, 6 (5.0%) were ELISA positive for STGO IgG, 2 (1.7%) were positive for TGR IgG, and 1 (0.8%) was positive for SFGR IgG. One serum sample was positive for IgG in TGR and SFGR ELISAs. For 6 samples positive by STGO ELISA, 4 also were positive for *O. tsutsugamushi* by Otsu47 qPCR, whereas 20 Otsu47-positive samples were STGO ELISA negative.

Of 302 enrolled patients, 103 (34.1%) had qPCR-confirmed scrub typhus, 12 (4.0%) had qPCR-confirmed murine typhus, and 2 (0.7%) cases could not be diagnosed beyond genus-specific Rick17b qPCR positive (i.e., *Rickettsia* spp.). Thirty-five patients (11.6%) were qPCR-negative but had eschars.

Table 3 gives demographic data, clinical features, and laboratory findings for 115 patients with confirmed scrub typhus (n = 103) and murine typhus (n = 12). There was no major difference in the mean age between these 2 groups, but we did not differences in the geographic distribution of each group. Of the 103 scrub typhus cases, the highest proportion were from Hanoi Province (34, 33.0%), HaiDuong (10, 9.7%), and PhuTho (10, 9.7%). Most scrub typhus patients reported rural residence (66, 64.1%). Among 34 scrub typhus patients from Hanoi Province, 19 (55.9%) reported living in rural areas. More men (n = 10) than women (n = 2) had murine typhus; most lived in Hanoi Province (10, 83.3%) and were from urban areas (8, 66.7%). Farming was the most common occupation reported among both groups, 52 (50.5%) for scrub typhus patients and 5 (41.7%) for murine typhus patients.

**Table 3:** Demographic information, signs and symptoms, and laboratory results for 115 patients with PCR-confirmed scrub typhus or murine typhus, northern Vietnam, March 2015–March 2017*

Category	qPCR-positive	Scrub typhus, n = 103	Murine typhus, n = 12	p value†
Age, y, mean ± SD	50.0 ± 15.8	50.0 ± 16.5	48.4 ± 8.6	0.709‡
Demographics				
Sex				
M	56 (48.7)	46 (44.7)	10 (83.3)	**0.014**
F	59 (51.3)	57 (55.3)	2 (16.7)	**0.001**
Residence				
Hanoi	44 (38.3)	34 (33.0)	10 (83.3)	**0.001**
Rural area	70 (60.9)	66 (64.1)	4 (33.3)	0.059
Illness during summer, April–August	80 (69.6)	72 (69.9)	8 (66.7)	1.0
Farmer	57 (49.6)	52 (50.5)	5 (41.7)	0.762
Treatment in previous hospitals	82 (71.3)	74 (71.8)	8 (66.7)	0.741
Prior antimicrobial drug treatment, n = 109	33 (30.3)	32 (33.0)	1 (8.3)	0.152
Symptoms at illness onset				
Headache	92 (80.0)	81 (78.6)	11 (91.7)	0.513
Myalgia	81 (69.9)	73 (70.9)	8 (66.7)	0.769
Retro-orbital pain	21 (18.3)	18 (17.5)	3 (25.0)	0.535
Sore throat	13 (11.3)	13 (12.6)	0	0.529
Cough	42 (36.5)	38 (36.9)	4 (33.3)	1.0
Nausea	30 (26.1)	28 (27.2)	2 (16.7)	0.718
Vomiting	21 (18.4)	21 (20.6)	0	0.228
Abdominal pain, n = 114	12 (10.5)	12 (11.8)	0	0.527
Diarrhea	18 (15.7)	15 (14.6)	3 (25.0)	0.664
Physical signs				
Congested skin	90 (78.3)	82 (79.6)	8 (66.7)	0.290
Conjunctivitis	74 (64.4)	66 (64.1)	6 (66.7)	1.0
Eschar	55 (48.8)	55 (53.4)	0	**0**
Rash	39 (33.9)	35 (34.0)	4 (33.3)	1.0
Lymphadenopathy, n = 114	19 (16.7)	19 (18.6)	0	0.212
Hepatomegaly	11 (9.6)	10 (9.7)	1 (8.3)	1.0
Splenomegaly	7 (6.1)	6 (5.8)	1 (8.3)	0.548
Edema	14 (12.2)	13 (12.6)	1 (8.3)	1.0
Rales	24 (20.9)	22 (21.4)	2 (16.7)	1.0
Decreased breath sounds, n = 114	16 (14.0)	16 (15.7)	0	0.212
Convulsion	10 (8.7)	10 (9.7)	0	0.596
Laboratory test results				
Erythrocytes, T/L, median (IQR)	4.28 (3.9–4.72)	4.25 (3.86–4.69)	4.34 (4.16–4.85)	0.392§
Leukocytes, g/L, median (IQR)	8.73 (6.7–11.1)	9.1 (6.62–11.17)	8.14 (6.9–10.2)	0.631§
Platelets, g/L, median (IQR)	123 (66–194)	128 (65–194)	96 (79.5–196.5)	0.902§
Platelet <100 g/L	49 (42.6)	43 (41.8)	6 (50.0)	0.759
Alanine aminotransferase >40 IU/L, n = 113	102 (90.3)	91 (89.2)	11 (100.0)	0.597
Aspartate aminotransferase ST >37 IU/L, n = 113	106 (93.8)	95 (93.1)	11 (100.0)	1.0
Total bilirubin >17 µmol/L, n = 69	27 (39.1)	24 (40.0)	3 (33.3)	1.0
Albumin <32 g/L, n = 80	39 (48.8)	36 (50.7)	3 (33.3)	1.0
Creatinine >120 µmol/L, n = 113	16 (14.2)	15 (14.9)	1 (8.3)	1.0
Procalcitonin >0.25 ng/mL, n = 101	80 (79.2)	69 (77.5)	11 (91.7)	0.451
C-reactive protein >12 mg/L, n = 93	85 (91.4)	75 (90.4)	10 (100)	0.592
Treatment				
Doxycycline, n = 114	72 (63.2)	63 (61.8)	9 (75.0)	0.839
Chloramphenicol, n = 114	2 (1.8)	2 (2.0)	0 (0.0)	0.839
Doxycycline and chloramphenicol, n = 114	30 (26.3)	28 (27.5)	2 (16.7)	0.839
Albumin transfusion, n = 113	12 (10.6)	12 (11.9)	0 (0.0)	0.357
Respiratory support, n = 111	29 (25.7)	27 (26.7)	2 (16.7)	0.728
Outcomes				
Fever before admission, d, median (IQR)	9 (7–11)	9 (7–11)	8.5 (5–10)	0.130§
Afebrile¶ ≤72 h after treatment began, n = 73	37 (50.7)	31 (49.2)	6 (60.0)	0.736
Median no. days to afebrile, n = 73 (IQR)	3 (3–5)	4 (3–5)	3 (3–5)	0.694§
Median no. days in hospital (IQR)	7 (5–10)	7 (5–10)	8 (7–8.5)	0.822§
Death or palliative discharge	5 (4.4)	5 (4.9)	0 (0.0)	1.0

*Values are no. (%), except as indicated. Bold text indicates statistical significance. IQR, interquartile range. †p value calculated using Fisher exact test, except where noted. ‡p value calculated using the analysis of variance test. §p value calculated using Mann-Whitney test. ¶No temperature recorded >37.5°C over a 24-h period.

Patients with confirmed rickettsial disease frequently reported headache, myalgia, and skin hyperaemia (≤78.6% for scrub typhus patients and 91.7% for murine typhus patients). Other symptoms, such as rash, sore throat, cough, nausea, vomiting, abdominal pain, diarrhea, dizziness, lymphadenopathy, hepatomegaly, splenomegaly, and edema, were less frequent in all groups. All cases with lymphadenopathy (n = 19) were confirmed to be scrub typhus.

The most common clinical features among 103 confirmed scrub typhus patients, other than fever, were headache (81, 78.6%), myalgia (73, 70.9%), skin hyperaemia (82, 79.6%), and conjunctivitis (66, 64.1%). Rash was seen in only 34.0% (35) of scrub typhus patients, lymphodenopathy in 18.6% (19), and hepatomegaly in 9.7% (10). Complications in scrub typhus patients were common and included altered mental status (10, 9.7%), jaundice or hyperbilirubinemia (24, 40%), and pulmonary pathology (rales) (22, 21.4%). Eschars were found in 53.4% (55) of scrub typhus cases, whereas eschars were not observed in murine typhus cases. Most scrub typhus and murine typhus patients had elevated liver aminotransferases, creatinine, procalcitonin, C-reactive protein, and bilirubin (Table 3). We found no differences in the clinical or laboratory findings between patients with confirmed murine typhus and scrub typhus, though it is noteworthy that no patients with murine typhus had lymphadenopathy. 

Patients did not immediately seek treatment, which delayed time from the onset of symptoms to treatment to a median of 9 days (IQR 7–11) for scrub typhus patients and 8.5 days (IQR 5–10) for murine typhus patients. After beginning treatment, 49.2% (31/63) of scrub typhus patients and 60% (6/10) of murine typhus patients were afebrile in <72hrs. Average length of hospital stays were 7 days (IQR 5–10) for scrub typhus patients and 8 days (IQR 7–8.5) murine typhus patients. Doxycycline was used to treat of 89.2% (91/102) of scrub typhus patients and 91.7% (11/12) murine typhus patients, and 30 patients (28 with scrub typhus and 2 with murine typhus) were treated with doxycycline plus chloramphenicol. All patients treated with antimicrobial drugs were afebrile within 3–5 days after beginning treatment, ≤3 days for murine typhus and ≤4 days for scrub typhus patients, and all recovered. 

Five deaths due to scrub typhus are reported in this study. One patient died in the hospital, and the other 4 patients were discharged from the hospital with a serious medical condition at the request of their family members and died shortly after being discharged. Three (60%) of these patients were ≥60 years of age, and all 5 experienced respiratory failure, hypotension, cardiovascular compromise, elevated aminotransferase levels, coagulation disorder during hospitalization, and markedly elevated serum ferritin levels (>1,500 µg/mL). One also had elevated lactate dehydrogenase (>1,000 U/L). Lung infiltrations and consolidation were the most common imaging findings. All 5 deaths were due to delay in time to appropriate care. 

Of the 185 patients with suspected rickettsial disease who tested negative by molecular methods (qPCR–), 35 had eschar (eschar+) and 150 did not (eschar–). We noted several significant differences when comparing the demographic data, clinical symptoms, and laboratory results between these 2 groups with unknown etiology (Appendix Table 1). However, when the eschar+/qPCR– group with unknown etiology (n = 35) was compared to the scrub typhus qPCR+ group (n = 103), they were found to be similar for all but two categories (Appendix Table 2). By definition, 1 difference was that the eschar+/qPCR– group was characterized by having an eschar and many individuals in the other group did not. The second difference was that the eschar+/qPCR– group was more likely to have been treated with antimicrobial drugs before enrollment in this study than the qPCR+ group. Treatment with antimicrobial drugs is known to rapidly decrease the levels of rickettsial bacteria below detectable levels in blood, which may explain why qPCR results were negative in the eschar+ patients. Moreover, the comparison of the scrub typhus group to the group with unknown etiology that was eschar–/qPCR– showed many significant differences and therefore likely represents diverse etiologies (Appendix Table 3). Collectively, these results indicate that the presence of eschar is an important sign for the diagnosis of scrub typhus in northern Vietnam.

## Discussion

This study contributes to accumulating evidence that rickettsial diseases are a common cause of fever in hospitalized patients in northern Vietnam and suggests a predominance of scrub typhus and a lower prevalence for murine typhus. Of the 302 patients with suspected rickettsial disease we enrolled, we diagnosed scrub typhus in 103 (34.1%) and murine typhus in 12 (4.0%). Rickettsioses peaked during the summer rainy season, when vector populations (e.g., ticks, fleas, and trombiculid mites) are most abundant and active and workers are in the rice fields. Previous studies have similarly reported seasonality that corresponds to summer months and rice harvesting (*11*,*12*). 

Among a subset of 121 patients with suspected rickettsiosis whose serum samples were tested, 6 (5.0%) showed prevalence of STGO IgG, 2 (1.7%) TGR IgG, and 1 (0.8%) SFGR IgG. This finding differs from what we previously reported for the prevalence of IgG against STGO (1.1%, 10/908), TGR (6.5%, 58/908), and SFGR (1.7%, 15/908) among healthy persons in the rural BaVi District and an urban area of Hanoi using the same ELISAs described here (*15*). In our previous study, 40% of participants were <20 years of age (*15*). In contrast, the patients investigated here were older. Scrub typhus incidence has been reported to peak in the fifth decade of life (*12*), with highest observed prevalence in adults 61–80 years of age (*21*). Thus, it is not surprising that the average age of qPCR+ scrub typhus cases and eschar+/qPCR– cases in this study was 50.3 years (Appendix Table 3). Note that our seroprevalence is lower than that described for suburban Bangkok, Thailand, and urban and rural Malang, Indonesia (*21*).

Of note, 4 of 6 qPCR-positive patients had IgG-positive acute-phase serum samples. The positive ELISA results did not appear to be associated with duration of fever before acute-phase blood collection. Two of the samples came from patients with fever of 5 days’ duration, suggesting they may have had preexisting *O. tsutsugamushi*–specific IgG. For 2 patients with onset of symptoms at 8 and 10 days, IgG positivity could have been due to the current infection, a previous infection, or both (*22*,*23*). We analyzed acute-phase serum samples for 20 Otsu47-positive samples, and all were negative by STGO ELISA. These examples highlight the importance of collecting paired acute- and convalescent-phase samples at least 14 days apart to serologically confirm the diagnosis of scrub typhus (*23*).

Our study showed a high proportion of eschars among scrub typhus patients (53.4%), and presence of eschar, a well-known clinical sign for scrub typhus (*13*), was 1 consideration used for defining suspect cases. The proportion of patients with eschar among our study population was similar to reports from northern (56.2%) and central (62.9%) Vietnam and from Laos and South Korea (*11*,*13*,*24*). The proportion of eschars among scrub typhus patients appears variable and has been reported ≤7% in children from Thailand (*25*). We did not recruit young children in this study, which may explain the higher proportion of scrub typhus patients with eschar reported here.

A recent study conducted in Quang Nam Province in central Vietnam demonstrated that the presence of eschar was the chief diagnostic indicator for scrub typhus (*13*). In that study, the authors used qPCR assays on DNA extracted from swab samples of patients’ eschar to identify scrub typhus. They recommended that eschar qPCR should be routinely considered for rickettsial diagnosis in the early phase of infection because it is easy to perform and has a high positive predictive value (*13*).

We identified 35 patients with an eschar and clinical symptoms consistent with scrub typhus but who tested negative for *O. tsutsugamushi* by qPCR on blood samples. These patients may have had true scrub typhus or spotted fever cases, despite the negative qPCR results. The sensitivity of qPCR for blood may have been inadequate to detect the small number of bacteria present, especially after antimicrobial therapy was commenced (*26*). Rickettsial DNA is known to disappear quickly from blood after start of appropriate antimicrobial drug therapy. The sensitivity of qPCR on eschar swabs has been reported as higher than for blood samples (18/20 eschar swabs were positive by qPCR vs. 5/20 blood samples from same patients [*13*]), at 85%–86% with specificity of 100% (*13*,*27*). The presence of rickettsiae in eschar and rash samples is not affected by previous antimicrobial drug treatment (*23*). Thus, for qPCR, the specimen of choice for eschar- or rash-producing rickettsioses is a swab (eschars) or biopsy (eschar or rash) (*23*).

In our study population, the most common symptoms among qPCR-positive patients included fever, headache, myalgia, conjunctivitis, and rash. Some patients also experienced cough, vomiting, nausea, abdominal pain, lymphadenopathy, hepatomegaly, and splenomegaly. Patients with scrub typhus had a higher frequency of lymphadenopathy, a well-known clinical sign of scrub typhus (*28*). Headache is frequent with other illnesses, and other studies also have found that clinical characteristics often are not helpful in identifying rickettsial infections. We also saw a high proportion of scrub typhus patients with conjunctivitis and rash in our study. Rash is a typical finding among patients suspected of having rickettsiosis and was among the inclusion criteria for our study. Rash is reported in ≈45%–50% of patients with scrub typhus (*29*,*30*). However, its frequency varies between areas, as seen in Japan (93%), Thailand (7%), and India (1.7%) (*31*–*33*). A few studies conducted in central Vietnam reported that the proportion of scrub typhus patients with rash was low (31.2%) or absent, as in a study in central Vietnam (*13*). Rash may be difficult to diagnose in patients with relatively dark skin, or may be absent at time of examination in the hospital. Most scrub typhus patients had fever for >7 days, time in which rash may have disappeared. We found no major differences in clinical manifestations between patients from Hanoi Province and other provinces of northern Vietnam, suggesting an absence of bias toward higher severity among patients traveling far to the hospital.

Complications of scrub typhus include jaundice, meningoencephalitis, myocarditis, acute respiratory distress, and renal failure (*34*,*35*). When treatment is delayed or does not include appropriate antimicrobial drugs, case-fatality rates are up to 6.0% but can be up to 70% (*29*). Our fatality rate of 4.9% (5/103) is similar to that in previous reports (*12*).

Murine typhus is a more challenging disease to diagnose clinically than scrub typhus because symptoms are nonspecific. The low number (n = 12) of confirmed murine typhus cases in our study makes it difficult to compare murine typhus with scrub typhus and other rickettsial diseases. However, patients with murine typhus experienced myalgia, conjunctivitis, nausea, cough, and vomiting less frequently than patients with scrub typhus, and no lymphadenopathy or eschars were described, which counters previous reports (*11*). Consistent with other reports, however, murine typhus was associated with elevated liver enzymes, procalcitonin, and C-reactive protein (*11*,*36*). These clinical findings may be useful for distinguishing the 2 diseases in areas where both are common (*24*).

Most scrub typhus and murine typhus patients in our study were treated with doxycycline, the recommended treatment for all ages. Chloramphenicol was used in severe cases as an additional drug. Within 72 hours of starting antimicrobial drug therapy, 49.2% (31/63) of scrub typhus and 60% (6/10) of murine typhus patients were afebrile. These data align with other reports (*11*). Rapid clinical improvement after using effective antimicrobial drugs is reported to back up clinical diagnoses of rickettsioses (*37*–*39*).

This study determined that scrub typhus was the predominant rickettsial disease diagnosed among patients hospitalized with acute undifferentiated fever in northern Vietnam. Public health awareness of scrub typhus and other rickettsial diseases in Vietnam should be enhanced to ensure healthcare providers can diagnose and treat these diseases successfully.

Appendix Additional information on clinical manifestations and molecular diagnosis of scrub typhus and murine typhus, Vietnam, 2015–2017.
