# Supplementary material for: Clinical Manifestations and Molecular Diagnosis of Scrub Typhus and Murine Typhus, Vietnam, 2015–2017
Source: Emerg Infect Dis. 2019 Apr;25(4):633–41. doi: 10.3201/eid2504.180691 (PMC6433017; doi:10.3201/eid2504.180691)
Supplement: Appendix — Additional information on clinical manifestations and molecular diagnosis of scrub typhus and murine typhus, Vietnam, 2015–2017. [file 18-0691-Techapp-s1.pdf]

Article DOI: https://doi.org/10.3201/eid2504.180691

Clinical Manifestations and Molecular Diagnosis of Scrub Typhus and Murine Typhus, Vietnam, 2015–2017

Appendix

Appendix Table 1. Demographic, clinical manifestations, and laboratory results for 185 patients with suspected rickettsial disease who tested negative with qPCR, 35 with eschar and 150 without eschar, northern Vietnam, 2015-2017*

	Total qPCR	Eschar–, qPCR–	Eschar+, qPCR–	p value†
Age (mean ± SD)	48 (34–59)	47.5 (34–59)	50 (38 – 65)	0.352‡
Demographic				
Male sex	101 (54.6)	83 (55.3)	18 (51.3)	0.676§
Living in Hanoi	77 (41.6)	66 (44.0)	24 (68.6)	0.174§
Living in rural areas	100 (54.1)	76 (50.7)	2 (20.0)	0.056§
Summer (April–August)	143 (77.3)	120 (80.0)	23 (65.7)	0.069§
High-exposure (farmer)	68 (36.8)	52 (34.7)	16 (45.7)	0.222§
Treatment in previous hospitals	122 (66.0)	96 (64.0)	26 (74.3)	0.248§
Prior antimicrobial drug treatment, n = 176	49 (27.8)	30 (21.0)	19 (57.6)	**0**
Sign and symptoms onset				
Fever, n = 184	184 (100)	149 (100)	35 (100)	–
Headache	140 (75.7)	109 (72.7)	31 (88.6)	**0.025**
Myalgia	102 (55.1)	76 (50.7)	26 (74.3)	**0.007**
Retro-orbital pain	21 (11.4)	12 (8.0)	9 (25.7)	**0.003**
Sore throat, n = 184	13 (11.5)	13 (12.6)	0 (0.0)	1.000
Cough	67 (36.2)	55 (36.7)	12 (34.3)	0.876
Nausea	30 (16.2)	19 (12.7)	11 (31.4)	**0.005**
Vomiting	24 (13.0)	17 (11.3)	7 (20.0)	0.203
Abdominal pain	15 (8.1)	14 (9.3)	1 (2.9)	0.547
Diarrhea, n = 184	25 (13.6)	20 (13.4)	5 (14.3)	1.0
Physical signs				
Congested skin, n = 184	107 (58.2)	78 (52.4)	29 (82.7)	**0.001**§
Conjunctivitis	75 (40.5)	59 (39.3)	16 (45.7)	0.489§
Rash	31 (16.8)	24 (16.0)	7 (20.0)	0.749
Lymphadenopathy	15 (8.1)	10 (6.7)	5 (14.3)	0.166
Hepatomegaly	11 (9.7)	10 (9.7)	1(10.0)	0.597
Splenomegaly, n = 184	6 (3.3)	6 (4.0)	0 (0.0)	0.487
Edema	11 (6.0)	6 (4.0)	5 (14.3)	**0.03**
Rales	27 (14.6)	22 (14.7)	5 (14.3)	0.954§
Decreased breath sound	13 (7.0)	10 (6.7)	3 (8.6)	0.714
Convulsion	10 (8.9)	10 (9.7)	0 (0.0)	**0.043**
Laboratory test¶				
Erythrocyte (T/L), median (P_{25}–P_{75}), n = 184	4.24 (3.67–4.74)	4.24 (3.98–4.74)	4.24 (3.83–4.71)	0.989‡
Leukocyte (g/L), median (P_{25}–P_{75}), n = 184	8.77 (5.81–11.3)	8.51 (6.1–11.3)	9 (5.1–12.6)	0.849‡
Platelet (g/L), median (P_{25}–P_{75}), n = 184	178.5 (103.5–250.5)	191 (116–257)	144 (79–193)	**0.009**‡
Platelet <100 g/L, n = 184	44 (23.9)	29 (19.5)	15 (42.9)	**0.004**§
ALT >40 IU/L, n = 175	126 (72.0)	101 (70.1)	25 (80.7)	0.237§
AST >37 IU/L, n = 179	128 (71.5)	100 (69.0)	28 (82.4)	0.120§
Total bilirubin >17µmol/L, n = 85	21 (24.7)	17 (22.7)	4 (40.0)	0.254
Albumin <32 g/L, n = 107	78 (72.9)	66 (76.7)	12 (57.1)	**0.07**§
Creatinine >120 µmol/L, n = 179	18 (10.1)	15 (10.3)	3 (8.8)	0.791§
PCT >0.25 ng/mL, n = 166	105 (63.3)	79 (58.1)	26 (86.7)	**0.003**§
CRP >12 mg/L, n = 156	127 (81.4)	105 (80.2)	22 (88.0)	0.574
Treatment and result				
Doxycycline, n = 181	115 (63.5)	91 (61.9)	24 (70.6)	**0.003**
Chloramphenicol, n = 181	6 (3.3)	4 (2.7)	2 (5.9)	**0.003**
Doxycycline and chloramphenicol, n = 181	10 (5.5)	5 (3.4)	5 (14.7)	**0.003**
Albumin transfusion, n = 183	12 (6.6)	8 (5.4)	4 (11.4)	0.248
Respiratory support, n = 184	20 (10.9)	11 (7.4)	9 (24.7)	0.003

	Total qPCR	Eschar–, qPCR–	Eschar+, qPCR–	p value†
Age (mean ± SD)	48 (34–59)	47.5 (34–59)	50 (38 – 65)	0.352‡
Outcome				
Fever before admission, median days (P_{25}–P_{75}), n = 184	9 (6–10.5)	8 (6–10)	10 (7–11)	0.649‡
Afebrile# ≤72 h after treatment began, n = 68	37 (50.7)	31 (49.2)	6 (60.0)	0.248
Median days to afebrile (P_{25}–P_{75}), n = 68	4 (3–5)	4 (3–5)	3 (2–4)	0.064‡
Median no. days in hospital (P_{25}–P_{75})	7 (5–10)	7 (5–10)	7 (5–10)	0.849‡
Death and palliative discharge	5 (2.7)	2 (1.3)	3 (8.6)	**0.048**

*Values relate to 185 patients and no. (%), except where otherwise indicated. P_{25}–$P_{75,}$ 25th–75th percentile. Bold text indicates statistical significance.
†Calculated using Fisher exact test, except where noted.
‡Calculated using Mann-Whitney test.
§Calculated using χ^2 test.
¶ALT, alanine aminotransferase; AST, aspartate aminotransferase; CRP, C-reactive protein; PCT procalcitonin.
#No temperature recorded >37.5°C, over a 24-h period.

Appendix Table 2. Comparison of demographic, clinical manifestations, and laboratory results for 138 patients with suspected scrub typhus, northern Vietnam, 2015-2017*

	Scrub typhus+ Eschar+, qPCR–	qPCR+	Scrub typhus– Eschar+, qPCR–	p value†
Age (mean ± SD)	50.3 ± 16.7	50.2 ± 16.5	50.6 ± 17.6	0.918‡
Demographic				
Male sex	64 (46.4)	46 (44.7)	18 (51.4)	0.448§
Living in Hanoi Province	45 (32.6)	34 (33.0)	11 (31.4)	0.863§
Living in rural area	90 (65.2)	66 (64.1)	24 (68.6)	0.630§
Summer (April–August)	95 (68.8)	72 (69.9)	23 (65.7)	0.644§
High-exposure (farmer)	68 (49.3)	52 (50.5)	16 (45.7)	0.626§
Treatment in previous hospitals	100 (72.5)	74 (71.8)	26 (74.3)	0.780§
Prior antimicrobial drug treatment, n = 130	51 (39.2)	32 (33.0)	19 (57.6	**0.043**§
Symptoms				
Fever	136 (98.6)	102 (99.0)	34 (1.0)	0.444
Headache	112 (81.2)	81 (78.6)	31 (88.6)	0.175
Myalgia	99 (71.7)	73 (70.9)	26 (74.3)	0.525
Retro-orbital pain	27 (19.6)	18 (17.5)	9 (25.7)	0.482
Sore throat	17 (12.4)	13 (12.6)	4 (11.8)	0.531
Cough	50 (36.2)	38 (36.9)	12 (34.3)	0.911
Nausea	39 (28.3)	28 (27.2)	11 (31.4)	0.858§
Vomiting, n = 137	28 (20.4)	21 (20.6)	7 (20.0)	1.0
Abdominal pain, n = 137	13 (9.5)	12 (11.8)	1 (2.9)	0.104
Diarrhea	20 (14.5)	15 (14.6)	5 (14.3)	0.648
Physical signs				
Congested skin	111 (80.4)	82 (79.6)	29 (82.7)	0.676§
Conjunctivitis	82 (59.4)	66 (64.1)	16 (45.7)	0.056§
Eschar	90 (65.2)	55 (53.4)	35 (100)	**0**§
Rash	42 (30.4)	35 (34.0)	7 (20.0)	0.144
Lymphadenopathy	0 (0.0)	19 (18.6)	0 (0.0)	0.359
Hepatomegaly	15 (10.9)	10 (9.7)	5 (14.3)	0.530
Splenomegaly	6 (4.4)	6 (5.8)	0 (0.0)	0.337
Edema	18 (13.0)	13 (12.7)	5 (14.3)	0.777
Rales	27 (19.6)	22 (21.4)	5 (14.3)	0.362§
Decreased breath sound, n = 137	19 (13.9)	16 (15.7)	3 (8.6)	0.400
Convulsion	14 (10.1)	10 (9.7)	4 (11.4)	0.752
Laboratory test¶				
Erythrocyte (T/L), median (P_{25}–P_{75})	4.25 (3.86–4.7)	4.25 (3.86–4.69)	4.24 (3.83–4.71)	0.760#
Leukocyte (g/L), median (P_{25}–P_{75})	9.05 (6.42–11.17)	9.1 (6.62–11.17)	9 (5.1–12.6)	0.828#
Platelet (g/L), median (P_{25}–P_{75})	129 (66–193)	128 (65–194)	144 (79–193)	0.701#
Platelet (g/L) <100 g/L	58 (42.0)	43 (41.8	15 (42.9)	0.909§
ALT >40 IU/L, n = 133	116 (87.2)	91 (89.2)	25 (80.7)	0.226
AST >37 IU/L, n = 136	123 (90.4)	95 (93.4)	28 (82.4)	0.089
Total bilirubin >17µmol/L, n = 70	28 (40.0)	24 (40.0)	4 (40.0)	1.0
Albumin <32 g/L, n = 92	39 (49.4)	36 (50.7)	3 (37.5)	0.527§
Creatinine >120 µmol/L, n = 135	18 (13.3)	15 (14.9)	3 (8.8)	0.561
PCT >0.25 ng/mL, n = 119	95 (79.8)	69 (77.5)	26 (86.7)	0.281§
CRP >12 mg/L, n = 108	97 (89.8)	75 (90.4)	22 (88.0)	0.714
Treatment and result				
Doxycycline, n = 136	87 (64.0)	63 (61.2)	24 (70.6)	0.285
Chloramphenicol, n = 136	4 (2.9)	2 (2.0)	2 (5.9)	0.285
Doxycycline and chloramphenicol, n = 136	33 (24.3)	28 (27.2)	5 (14.7)	0.285
Albumin transfusion, n = 136	16 (11.9)	12 (12.0)	4 (11.8)	1.0
Respiratory support, n = 136	36 (26.5)	27 (26.7)	9 (25.7)	0.906§
Outcome				
Fever before admission, median days (P_{25}–P_{75})	9 (7–11)	9 (7–11)	10 (7–11)	0.908#
Afebrile** ≤72 h after treatment began, n = 78	40 (51.3)	31 (49.2)	9 (60.0)	0.452§
Median days to afebrile (P_{25}–P_{75}), n = 78	3 (2–5)	4 (3–5)	3 (2–4)	0.097#
Median no. days in hospital (P_{25}–P_{75})	7 (5–10)	7 (5–10)	7 (5–10)	0.945#
Death and Palliative discharge	8 (5.8)	5 (4.9)	3 (8.6)	0.418

*Values relate to 138 patients (103 patients with confirmed scrub typhus, eschars, and positive qPCR; 35 patients with negative qPCR eschar) and no. (%), except where otherwise indicated. P_{25}–$P_{75,}$ 25th–75th percentile. Bold text indicates statistical significance.
†Calculated using Fisher exact test, except where noted.
‡Calculated using the analysis of variance test.
§Calculated using χ^2 test.
¶ALT, alanine aminotransferase; AST, aspartate aminotransferase; CRP, C-reactive protein; PCT procalcitonin.
#Calculated using Mann-Whitney test.
**No temperature recorded >37.5°C, over a 24-h period.

Appendix Table 3. Clinical manifestations and laboratory results for 253 patients, 103 with qPCR-confirmed (qPCR+) scrub typhus and 150 with eschar-negative, qPCR-negative (eschar–, qPCR–) results, northern Vietnam, 2015-2017*

Category	Scrub Typhus eschar–, qPCR–	Scrub Typhus	Eschar–, qPCR–	p value†
Symptoms onset				
Fever, n = 252	252 (100)	102 (99.0)	149 (100)	0.407
Headache	190 (75.1)	81 (78.6.)	109 (72.7)	0.467
Myalgia	149 (58.9)	73 (70.9)	76 (50.7)	**0.002**
Retro-orbital pain, n = 113	30 (11.9)	18 (17.5)	12 (8.0)	**0‡**
Sore throat	30 (11.9)	13 (12.6)	17 (11.3)	0.139
Cough	93 (36.8)	38 (36.9)	55 (36.7)	0.720
Nausea	47 (18.6)	28 (27.2)	19 (12.7)	**0.002**
Vomiting, n = 252	38 (15.1)	21 (20.6)	17 (13.1)	**0.023**
Abdominal pain, n = 252	26 (10.3)	12 (11.8)	14 (9.3)	0.097
Diarrhea, n = 252	35 (13.9)	15 (14.6)	13 (14.2)	0.236
Physical signs				
Congested skin, n = 252	160 (63.5)	82 (79.6)	78 (52.4)	**0‡**
Conjunctivitis	149 (49.4)	66 (64.1)	59 (39.3)	**0‡**
Eschar	55 (21.7)	55 (53.4)	0 (0.0)	**0‡**
Rash	59 (23.3)	35 (34.0)	24 (16.0)	**0.001**
Lymphadenopathy, n = 251	25 (10.0)	19 (18.6)	6 (4.0)	**0‡**
Hepatomegaly	20 (7.9)	10 (9.7)	10 (6.7)	0.378‡
Splenomegaly	12 (4.8)	6 (5.8)	6 (4.3)	0.556
Edema	19 (7.5)	13 (12.7)	6 (4.0)	**0.011‡**
Rales	44 (17.39)	22 (21.4)	22 (14.67)	0.168‡
Decreased breath sound, n = 252	26 (10.3)	16 (15.7)	10 (6.7)	**0.021‡**
Convulsion	14 (5.5)	10 (9.7)	4 (2.7)	**0.016‡**
Laboratory test§				
Erythrocyte (T/L), median (P_{25}–P_{75}), n = 252	4.25 (3.95–4.73)	4.25 (3.86–4.69)	4.24 (3.98–4.74)	0.589¶
Leukocyte (g/L), median (P_{25}–P_{75}), n = 252	8.72 (6.32–11.19)	9.1 (6.62–11.17)	9.1 (6.1–11.3)	0.455¶
Platelet (g/L), median (P_{25}–P_{75}), n = 252	163 (86.9–244)	128 (65–194)	191 (116–257)	**0¶**
Platelet <100 (g/L), n = 252	49 (43.4)	43 (41.8	6 (0.0)	0.070‡
ALT >40 IU/L, n = 246	192 (78.1)	91 (89.2)	101 (70.1)	**0‡**
AST >37 IU/L, n = 247	195 (79.0)	95 (93.4)	100 (67.0)	**0‡**
Total bilirubin >17µmol/L, n = 135	41 (30.4)	24 (40.0)	17 (22.7)	**0.030‡**
Albumin <32 g/L, n = 157	56 (35.7)	36 (50.7)	20 (23.3)	**0‡**
Creatinine >120µmol/L, n = 111	30 (12.2)	15 (14.9)	15 (10.3)	0.288‡
PCT >0.25 ng/mL, n = 225	148 (65.8)	69 (77.5)	79 (58.1)	**0.003‡**
CRP >10 mg/L, n = 214	180 (84.1)	75 (90.4)	105 (80.2)	**0.047‡**

*Values relate to 253 patients and no. (%), except where otherwise indicated. P_{25}–P_{75}, 25th–75th percentile. Bold text indicates statistical significance.
†Calculated using Fisher exact test, except where noted.
‡Calculated using χ^2 test.
§ALT, alanine aminotransferease; AST, aspartate aminotransferase; CRP, C-reactive protein; PCT procalcitonin.
¶Calculated using Mann-Whitney test.

Appendix Table 4. Comparison of demographic, clinical manifestations, and laboratory results for 103 patients with scrub typhus from Hanoi Province (n = 34) and other provinces (n = 69), northern Vietnam, 2015-2017*

	All scrub typhus cases	Hanoi	Other Provinces	p value†
Age, y (mean ± SD)	50.2 ± 16.5	52.2 ± 15.3	49.2 ± 17.1	0.390‡
Transferred from other hospitals or clinics	74 (71.8)	16 (47.1)	58 (84.1)	**0**§
Antimicrobial drug treatment before admission, n = 97	32 (33.0)	6 (20.0)	26 (38.8)	0.143§
Signs and symptoms				
Headache	81 (78.6)	26 (76.5)	55 (79.7)	0.459
Myalgia	73 (70.9)	24 (70.6)	49 (71.0)	1.0
Retro-orbital-pain	18 (17.5)	8 (23.5)	10 (14.5)	0.338§
Sore throat	13 (12.6)	4 (11.8)	9 (13.0)	1.0
Cough	38 (36.9)	10 (29.4)	28 (40.5)	0.402
Nausea	28 (27.2)	8 (23.5)	20 (29.0)	0.702
Vomiting	21 (20.6)	5 (14.7)	16 (23.5)	0.535
Abdominal pain	12 (11.8)	3 (8.8)	9 (13.2)	0.914
Physical signs				
Congested skin	82 (79.6)	26 (76.5)	56 (81.2)	0.579§
Conjunctivitis	66 (64.1)	22 (64.7)	44 (63.8)	0.926§
Eschar	55 (53.4)	17 (50.0)	38 (55.1)	0.627§
Rash	35 (34.0)	11 (32.4)	24 (34.8)	0.930
Lymphadenopathy, n = 102	18 (17.7)	3 (8.8)	15 (22.1)	0.136
Hepatomegaly	10 (9.7)	1 (2.9)	9 (13.0)	0.159
Splenomegaly	6 (5.8)	1 (2.9)	5 (7.3)	0.661
Edema	13 (12.6)	2 (5.9)	11 (15.9)	0.211
Rales	22 (21.4)	7 (20.6)	15 (21.7)	0.893§
Decreased breath sound, n = 102	16 (15.7)	4 (11.8)	12 (17.7)	0.441§
Convulsion	10 (9.7)	3 (8.8)	7 (10.1)	1.0
Laboratory test¶				
Platelet <100 g/L	43 (41.8)	14 (41.2)	29 (42.0	0.934§
ALT >40 IU/L, n = 102	91 (89.2)	31 (93.9)	60 (87.0)	0.496
AST >37 IU/L, n = 102	95 (93.1)	31 (93.4)	69 (92.8)	1.0
Bilirubin Total>17µmol/L, n = 60	36 (60.0)	12 (57.1)	24 (61.5)	0.740
Albumin <32 g/L, n = 71	36 (50.7)	10 (40.0)	26 (56.5)	0.220
Creatinine >120 µmol/L, n = 101	15 (14.9)	3 (9.1)	12 (17.7)	0.374
PCT >0.25 ng/mL, n = 89	69 (77.5)	19 (73.1)	50 (79.4)	0.218§
CRP >12 mg/L, n = 92	75 (90.4)	25 (83.3)	50 (94.3)	0.131
Treatment and result				
Doxycycline	63 (61.2)	21 (61.7)	42 (60.9)	0.634
Chloramphenicol	2 (1.9)	0 (0.0)	2 (2.9)	0.634
Doxycycline and chloramphenicol	28 (27.2)	11 (32.4)	17 (24.6)	0.634
Albumin transfusion, n = 101	12 (11.9)	5 (14.7)	7 (10.5)	0.532
Respiratory support, n = 101	27 (26.7)	8 (23.5)	19 (28.4)	0.604§
Outcome				
Fever before admission, median days (P_{25}–P_{75})	9 (7–11)	9 (7–11)	9 (7–11)	0.783**
Median no. days to afebrile# (P_{25}–P_{75}), n = 63	4 (3–5)	4 (2.5–5.5)	3 (3–5)	0.804**
Median no. days in hospital (P_{25}–P_{75})	7 (5–10)	7 (5–10)	7 (5–10)	0.797**
Palliative discharge and death	5 (4.9)	1 (2.9)	4 (5.8)	0.464

*Values relate to 103 patients and no. (%), except where otherwise indicated. P_{25}–$P_{75,}$ 25th–75th percentile. Bold text indicates statistical significance.
†Calculated using Fisher exact test, except where noted.
‡Calculated using the analysis of variance test.
§Calculated using χ^2 test.
¶ALT, alanine aminotransferase; AST, aspartate aminotransferase; CRP, C-reactive protein; PCT procalcitonin.
#No temperature recorded >37.5°C, over a 24-h period.
**Calculated using Mann-Whitney test.
